# Appointments in the St. Peter’s Hospital, Albany, N. Y.

**Published:** 1870-12

**Authors:** 


### Appointments in the St. Peter's Hospital, Albany, N. Y.

Some of our readers will be interested in noticing the appointments in the St. Peter's Hospital, Albany, mainly, on account of difficulties heretofore placed before the profession.

Medical Officers.—Consulting Physician: Thomas Hun, M. D., 21 Elk

St. Attending Physicians: Edward Hun, M. D., 23 Elk St.; S. O. Vanderpoel, M. D., cor. State and Eagle Streets; Chas. H. Porter, M. D., 55 Eagle Street; Diseases of the Eye and Ear: C. A. Robertson, M. D., 17 Washington Avenue. Attending Surgeons: Dan'l M. Stimson, M. D., 17 Fayette Street; J. R. Boulware, M. D., cor. Hamilton and Eagle Streets; Diseases of Women: J. V. P. Quackenbush, M. D., 712 Broadway; Resident Physician: Caleb Lyon.

